# Time-advanced occurrence of moderate-size earthquakes in a stable intraplate region after a megathrust earthquake and their seismic properties

**DOI:** 10.1038/s41598-018-31600-5

**Published:** 2018-09-06

**Authors:** Tae-Kyung Hong, Junhyung Lee, Seongjun Park, Woohan Kim

**Affiliations:** 1 Yonsei University, Department of Earth System Sciences, 50 Yonsei-ro, Seodaemun-gu Seoul, 120-749 South Korea; 2 Gyeongsang National University, Department of Earth and Environmental Sciences and RINS, Jinju, Gyeongsangnam-do, 660-701 South Korea

## Abstract

The distance-dependent coseismic and postseismic displacements produced by the 2011 *M*_*W*_9.0 Tohoku-Oki megathrust earthquake caused medium weakening and stress perturbation in the crust around the Korean Peninsula, increasing the seismicity with successive *M*_*L*_5-level earthquakes at the outskirts of high seismicity regions. The average *M*_*L*_5-level occurrence rate prior to the megathrust earthquake was 0.15 yr^−1^ (0.05–0.35 yr^−1^ at a 95% confidence level), and the rate has increased to 0.71 yr^−1^ (0.23–1.67 yr^−1^ at a 95% confidence level) since the megathrust earthquake. The 2016 *M*_*L*_5-level midcrustal earthquakes additionally changed the stress field in adjacent regions, inducing the 15 November 2017 *M*_*L*_5.4 earthquake. The successive 2016 and 2017 moderate-size earthquakes built complex stress fields in the southeastern Korean Peninsula, increasing the seismic hazard risks in the regions of long-term stress accumulation. The increased seismic risks may continue until the medium properties and stress field are recovered.

## Introduction

**Figure 1:**
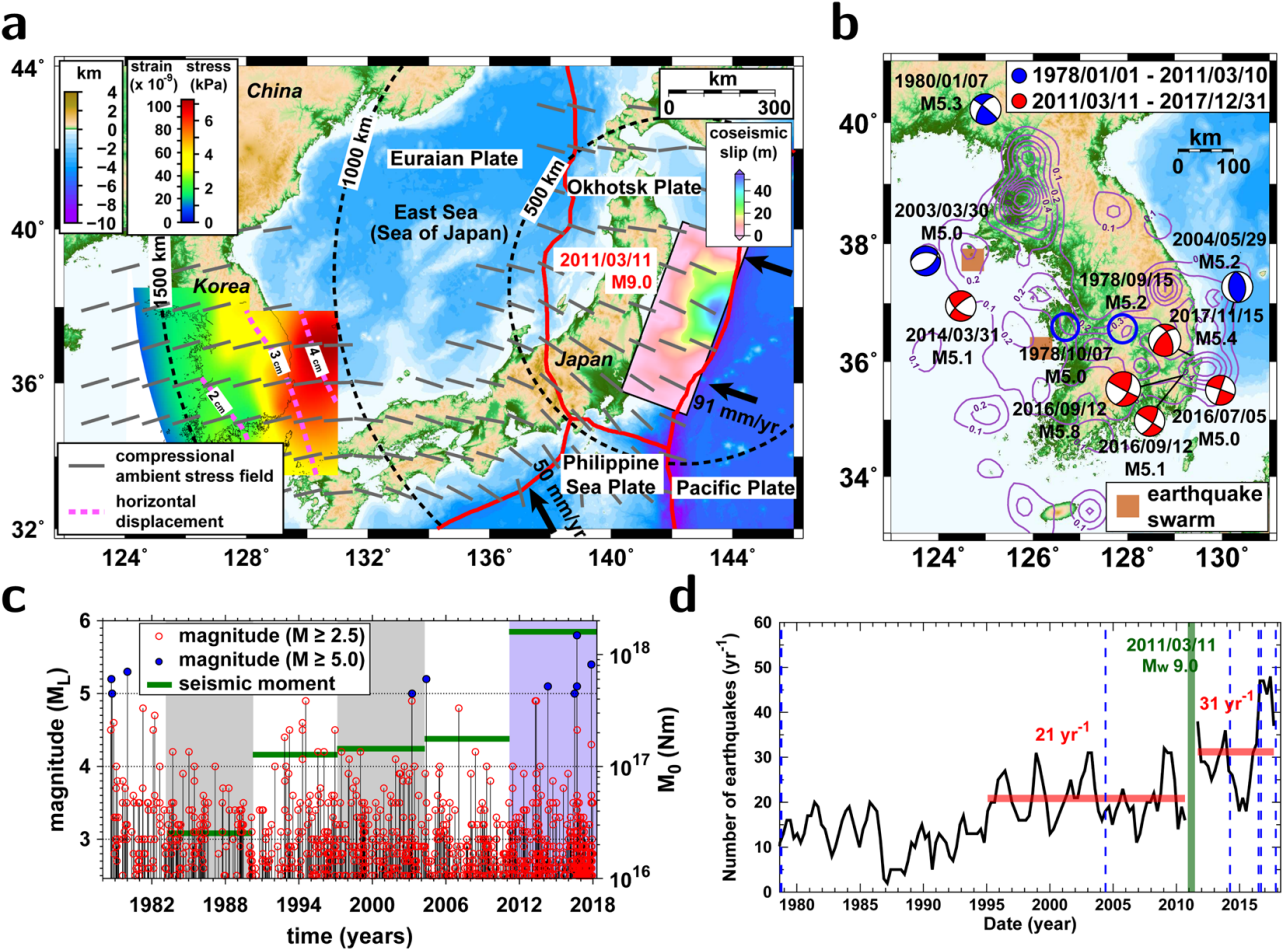
Moderate-size earthquake induction on the Korean Peninsula. (**a**) Map around the Korean Peninsula with a slip model of the 2011 *M*_*W*_9.0 Tohoku-Oki megathrust earthquake. The primary compression field is presented with solid bars^6^. The tension field and coseismic displacements incurred by the megathrust earthquake over the Korean Peninsula are presented^11^. The coseismic slip during the megathrust earthquake is presented on the map^10^. (**b**) Earthquakes with magnitudes greater than or equal to *M*_*L*_5.0 since 1978. The seismicity densities of instrumentally recorded earthquakes since 1978 are presented with contours^35^. The locations of earthquake swarms since the 2011 megathrust earthquake are marked on the map. (**c**) Earthquake occurrence since 1978 in the Korean Peninsula. Events with magnitudes greater than or equal to 5.0 are indicated. The total seismic moments emitted by the earthquakes every seven years are shown. The emitted seismic moments increased after the megathrust earthquake. (**d**) Temporal variation in yearly numbers of earthquakes with magnitudes greater than or equal to 2.5 from a declustered catalog. The average number of earthquakes was 21 yr^−1^ before the 2011 Tohoku-Oki megathrust earthquake and 31 yr^−1^ after the earthquake. The dates of earthquakes with magnitudes of *M* ≥ 5.0 are marked with broken lines. The seismicity increased abruptly after the megathrust earthquake. The figure was created using GMT 4.5.14 (https://www.soest.hawaii.edu/gmt/) and Adobe Illustrator CS6 (https://www.adobe.com/kr/products/illustrator.html).

The Korean Peninsula is located in a stable intraplate region of the eastern Eurasian plate. The continental crust lies in the peninsula and the Yellow Sea. A transitional crust between continental and oceanic crusts has developed in the East Sea (Sea of Japan)^1–5^. The region around the peninsula is under the influence of a compressional stress field with an ENE-directional compression and SSE-directional tension^6,7^ (Fig. 1). The instrumentally recorded earthquakes since 1978 indicated mild and diffused seismicity (Fig. 1).

However, the historical literature indicates large seismic damage on the Korean Peninsula. The largest seismic intensity reached IX on the modified Mercalli intensity (MMI) scale^8^. The historical seismicity displays high similarity with the instrumentally recorded seismicity over most regions in the Korean Peninsula, except the central peninsula around the Seoul metropolitan area^8,9^. The historical earthquake records present high seismicity with large earthquakes in the central peninsula.

The 2011 *M*_*W*_9.0 Tohoku-Oki megathrust earthquake occurred ~1200 km from the Korean Peninsula. The megathrust earthquake incorporated large lithospheric displacements up to regional distances^10–12^. The 2011 Tohoku-Oki megathrust earthquake produced coseismic displacements of ~4 cm around the east coast and ~2 cm around the west coast of the Korean Peninsula (Fig. 1)^11,13,14^. Comparable strengths of postseismic displacements followed the coseismic displacements for more than 3 years^12,15^.

The distance-dependent displacements produced tension stress over the Korea Peninsula^11^ (Fig. 1), and the discriminative crustal extension decreased the seismic velocity in the crust^12,16–19^. The seismicity increased abruptly after the megathrust earthquake, and unusual episodic earthquake swarms were observed for 60 days after 29 April 2013 and 120 days after 2 June 2013 at two regions in the Yellow Sea (Fig. 1)^11^. The seismicity increase may have been caused by the decreasing yield strength of the medium due to instantaneous activation of the tension field by crustal extension^11^. It was suggested that a small change in stress field may induce significant seismicity changes^20^. The earthquakes occurred in both high seismicity regions and low seismicity regions, suggesting that the earthquakes were fostered in the low seismicity region^11,21^.

The stress field inferred from the focal mechanism solutions of earthquakes after the 2011 Tohoku-Oki megathrust earthquake is close to the ambient stress field inferred from the focal mechanism solutions of earthquakes before the 2011 Tohoku-Oki megathrust earthquake. The compression-axis directions of the 12 September 2016 *M*_*L*_5.8 earthquake and its aftershocks were N70°E to N77°E^22^. The maximum compression-axis directions of the ambient stress field before the megathrust earthquake were ~N77°E (±1.2° at a 95% confidence level)^6^. Small changes in the stress field and medium properties produce earthquakes with new fault planes whose orientations conform to the stress field^11^. Earthquakes with different faulting types occurred after the megathrust earthquake^11^, possibly due to abrupt changes in differential stresses that develop new fault planes with different faulting behavior^23,24^.

Ten *M*_*L*_5-level earthquakes have occurred on the Korean Peninsula since 1978, when national seismic monitoring was commenced (Fig. 1). The *M*_*L*_5-level earthquakes were generally scattered around the suburbs of high seismicity regions (Fig. 1). The number of *M*_*L*_5-level earthquakes has increased since the 2011 Tohoku-Oki megathrust earthquake: one-half of the moderate-size earthquakes (5 events) occurred since the 2011 Tohoku-Oki megathrust earthquake. We investigate the properties of the successive *M*_*L*_5-level earthquakes and their induction mechanisms on the Korean Peninsula.

## Results

### Seismicity change

The seismic velocities in the crust of the Korean Peninsula decreased by ~3% instantly after the 2011 Tohoku-Oki megathrust earthquake^12^. Seismic velocities are recovered with time^12^. We examine the seismicity changes by declustering the seismicity, which excludes the aftershocks from the earthquake catalog (Fig. 1) (see supplementary materials). The earthquake catalog since 1978 is complete for seismicity with magnitudes greater than or equal to *M*_*L*_2.5^9,11^ (see supplementary materials). The declustered seismicity of earthquakes with *M*_*L*_ ≥ 2.5 presents seismicity rates of 21 yr^−1^ (18.72–23.25 yr^−1^ at a 95% confidence level) prior to the megathrust earthquake and 31 yr^−1^ (27.13–35.53^−1^ at a 95% confidence level) since the megathrust earthquake (Fig. 1).

The increase in seismicity rate was caused an increase in seismic energy emission. The seismic moments emitted from the earthquakes on the Korean Peninsula for 82 months since the 2011 Tohoku-Oki megathrust earthquake were more than 10 times larger than those before the megathrust earthquake for the same time duration (Fig. 1).

The seismicity before the megathrust earthquake may be a consequence of tectonic loading in the crust of the Korean Peninsula. The average occurrence rate of the ten *M*_*L*_5-level earthquakes for the 40 years of 1978–2018 is 0.25 yr^−1^ (0.12–0.46 yr^−1^ at a 95% confidence level). The occurrence rate prior to the megathrust earthquake, 0.15 yr^−1^ (0.05–0.35 yr^−1^ at a 95% confidence level) changed to 0.71 yr^−1^ (0.23–1.67 yr^−1^ at a 95% confidence level) after the megathrust earthquake. The probabilities of having five *M*_*L*_5-level earthquakes in the 7 years since the megathrust earthquake are less than 3% and 1% for occurrence rates of 0.25 yr^−1^ and 0.15 yr^−1^ in terms of Poissonian earthquake occurrence^25,26^ (see supplementary materials). This observation suggests that the recent *M*_*L*_5-level earthquakes since the 2011 megathrust earthquake may be time-advanced events that might otherwise have occurred at later times^27^.

The first *M*_*L*_5-level earthquake (*M*_*L*_5.1) since the megathrust earthquake occurred on 31 March 2014 in the Yellow Sea. An *M*_*L*_5.0 earthquake occurred in a region off the southeastern coast on 5 June 2016. Midcrustal *M*_*L*_ 5.1 and 5.8 earthquakes occurred within a time interval of 48 min on the southeastern Korean Peninsula on 12 September 2016^22^. The *M*_*L*_5.8 earthquake was the largest event in the instrumental seismic monitoring history since 1978. The event occurred in a midcrustal blind fault^22^, and it released the stress of the fault to adjacent regions, producing aftershocks. The aftershocks occurred dominantly for more than 1 year in regions with positive Coulomb stress changes.

The 15 November 2017 *M*_*L*_5.4 earthquake occurred in a region of positive Coulomb stress changes by the 2016 *M*_*L*_5-level earthquakes^22^. The fault planes of the moderate-size events in 2016 and 2017 were not found on the surface. The 2016 and 2017 moderate-size earthquakes both produced strong seismic waves, presenting similar spatial distributions of ground motion over the Korean Peninsula (Fig. 1). The peak seismic intensities of the events reached VIII on the modified Mercalli intensity (MMI) scale. The 2016 *M*_*L*_5.8 earthquake yielded relatively stronger energy at frequencies of 1–4 Hz, while the 2017 *M*_*L*_5.4 earthquake yielded large energy at frequencies of 0.15–0.5 Hz (Fig. 1).

### Time-advanced earthquakes

We investigate the properties of time-advanced earthquakes in a stable intraplate region from the three inland *M*_*L*_5-level earthquakes in 2016 and 2017. The two consecutive *M*_*L*_5-level (*M*_*L*_5.1, 5.8) midcrustal earthquakes on 12 September 2016 perturbed the local stress field significantly^22^. Earthquakes started to occur in the regions of positive Coulomb stress changes induced by the 2016 moderate-size earthquakes (see supplementary materials).

The 2017 *M*_*L*_5.4 earthquake region was seismically quiescent before the 2011 Tohoku-Oki megathrust earthquake (see supplementary materials). Note that no events with magnitudes greater than or equal to 2.0 were reported in the epicenter region with a radius of 10 km before the 2016 *M*_*L*_5-level earthquakes. Four earthquakes with magnitudes greater than or equal to 2.0 (*M*_*L*_2.0, 2.2, 2.3, 3.1) occurred around the epicentral region with a radius of 3 km since the 2016 *M*_*L*_5-level earthquakes.

**Figure 2:**
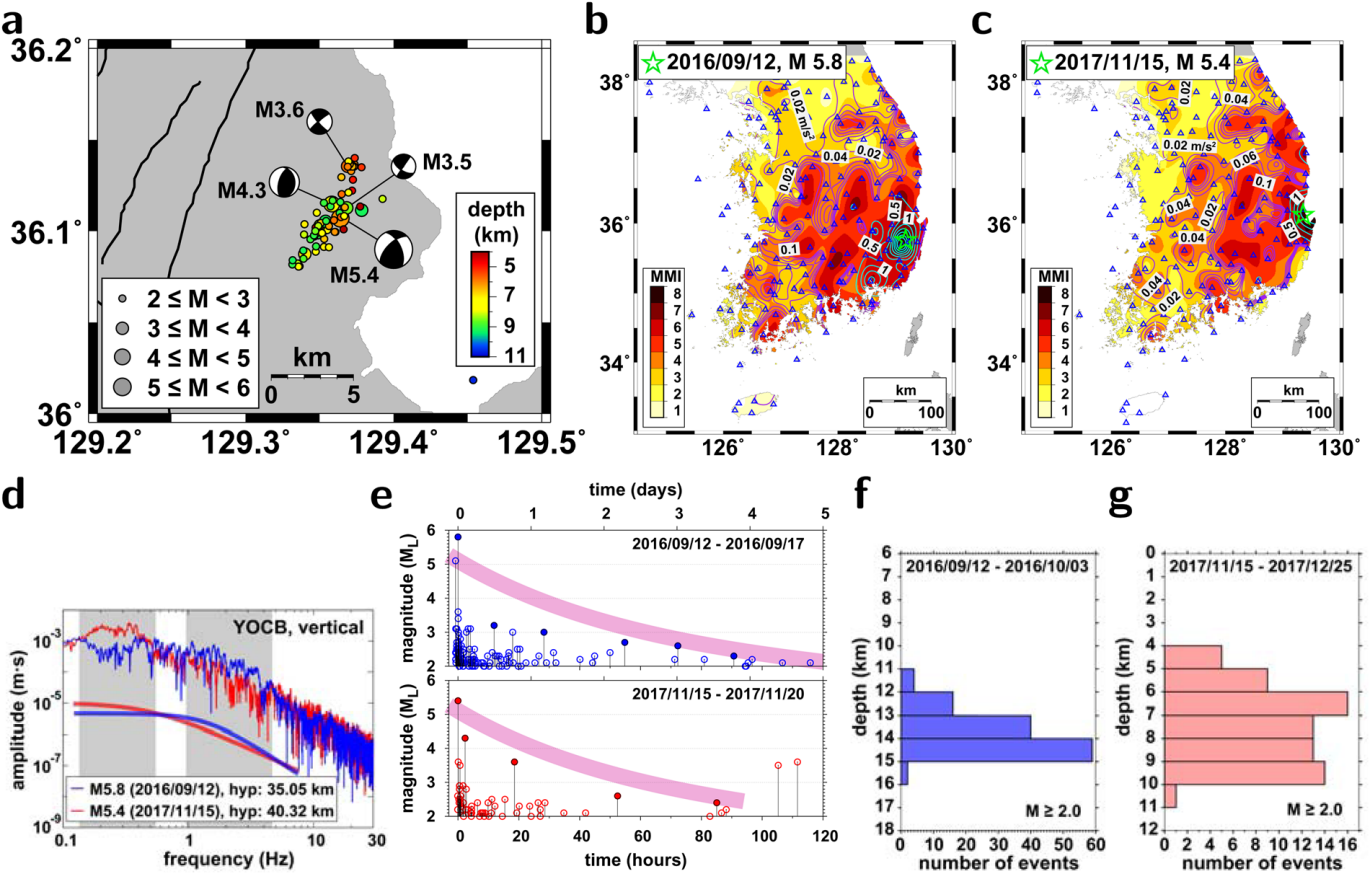
Seismic properties of two moderate-size earthquakes. (**a**) Spatial distribution of the 15 November 2017 *M*_*L*_5.4 earthquake and its aftershocks. Focal mechanism solutions of major earthquakes with the focal depths indicated. The events display different types of faulting. Peak ground accelerations in m/s and seismic intensities in the modified Mercalli intensity (MMI) scale of (**b**) the 15 November 2017 *M*_*L*_5.4 earthquake and (**c**) the 12 September 2016 *M*_*L*_5.8 earthquake. The seismic stations are marked with triangles. The spatial distributions of the seismic intensities of the events are comparable. (**d**) Comparison of the vertical displacement spectra of the 2017 *M*_*L*_5.4 earthquake and the 2016 *M*_*L*_5.8 at comparable distances at a common station (YOCB). The displacement spectra of the 2017 *M*_*L*_5.4 earthquake are rich in the low frequencies of 0.15–0.5 Hz. The 2016 *M*_*L*_5.8 earthquake displays relatively strong energy in the high frequencies of 1–4 Hz. (**e**) Aftershock occurrence with time after the 2016 *M*_*L*_5.8 earthquake and the 2017 *M*_*L*_5.4 earthquake. Comparison of focal depths of aftershocks between (**f**) the 2016 *M*_*L*_5.8 earthquake and (**g**) the 2017 *M*_*L*_5.4 earthquake. The aftershocks of the 2017 *M*_*L*_5.4 earthquake occur at shallower depths than those of the 2016 *M*_*L*_5.8 earthquake. The figure was created using GMT 4.5.14 (https://www.soest.hawaii.edu/gmt/) and Adobe Illustrator CS6 (https://www.adobe.com/kr/products/illustrator.html).

The seismic moment of the 2017 *M*_*L*_5.4 earthquake was 1.85 × 10^17^ Nm, and the corresponding moment magnitude was *M*_*w*_5.5 (see supplementary materials). The strike of the fault was N45°E, and the dip was 61° to the west. The fault for the 2016 *M*_*L*_5.8 earthquake presented a strike of N27°E and dip of 65° to the east^22^. The refined focal depth of the 2017 *M*_*L*_ 5.4 earthquake was 6.2 km. The earthquake showed a combined motion sense of reverse and strike-slip faulting (Fig. 2). The seismic energy was composed of a double-couple component (67%) and compensated linear vector dipole (CLVD) component (33%). This observation suggests a complex fault geometry and rupture.

The aftershock occurrence rate of the 2017 *M*_*L*_5.4 earthquake was smaller than that of the 2016 *M*_*L*_5.8 earthquake. The aftershocks of the 2016 *M*_*L*_5.8 earthquake lasted for more than one year. The number of aftershocks of the 2017 *M*_*L*_5.4 earthquake decreased rapidly with time (Fig. 2). However, the magnitudes of the aftershocks decreased with time at similar decay rates. The aftershocks of the 2017 *M*_*L*_5.4 earthquake were located at depths between 4 and 11 km, while those of the 2016 *M*_*L*_5.8 earthquake were distributed at depths between 11 and 16 km^22^ (Fig. 2). The focal depths of the mainshocks and aftershocks of the 2016 *M*_*L*_5.8 earthquake and the 2017 *M*_*L*_5.4 earthquake were within the typical seismicity depths (4–20 km) on the Korean Peninsula^28^.

The seismic energy from the 2017 *M*_*L*_5.4 earthquake was rich in low frequencies around ~0.5 Hz compared to that from the 2016 *M*_*L*_5.8 earthquake (Fig. 2)^22^. The levels of displacement spectra of the 2016 *M*_*L*_5.8 earthquake and the 2017 *M*_*L*_5.4 earthquake at a common station at comparable distances were similar, despite an apparent magnitude difference in the local magnitude scale. The spatial distribution of the peak ground accelerations (PGAs) of the 2017 *M*_*L*_5.4 earthquake was similar to that of the 2016 *M*_*L*_5.8 earthquake (Fig. 2). The PGA decays gradually with distance on the Korean Peninsula^29^. The characteristic slow distance-dependent decay of PGAs suggests a high potential for seismic damage over wide regions when a large event occurs in the stable intraplate region.

The ground motions were particularly strong in the Quaternary sedimentary basin (Gyeongsang Basin) of the southeastern Korean Peninsula. The epicenter region of the 2017 *M*_*L*_5.4 earthquake is covered by a Tertiary sedimentary layer that causes seismic amplification. Significant liquefaction around the epicentral region was reported, which was not common in the earthquakes of the Korean Peninsula. The property damage from the 2017 *M*_*L*_5.4 earthquake was much higher than that from the 2016 *M*_*L*_5.8 earthquake. This difference may be partly due to the low-frequency-rich ground motions and surface sedimentary layer of the epicentral region in the 2017 *M*_*L*_5.4 earthquake.

### Blind faults incurring time-advanced earthquakes

**Figure 3:**
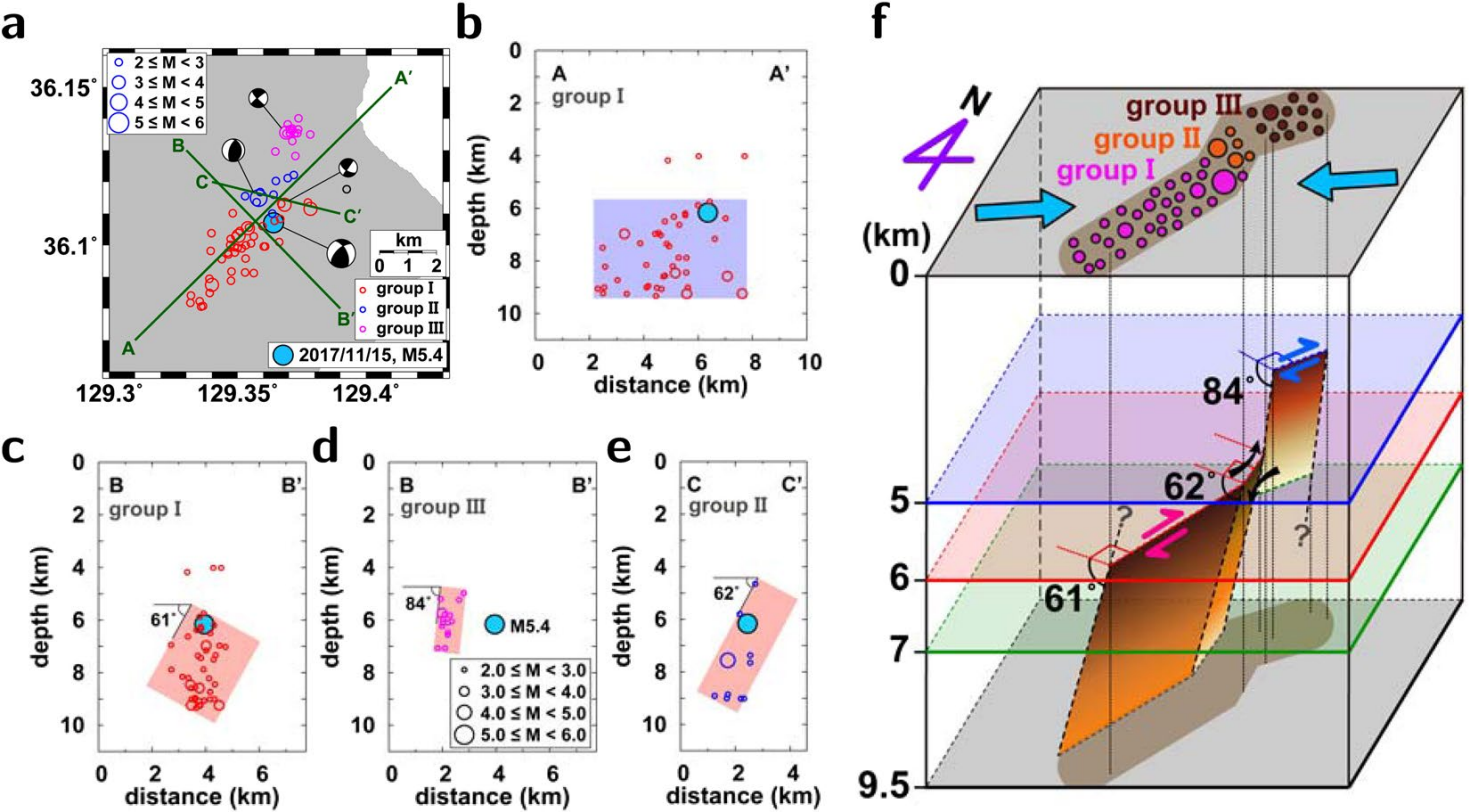
The 2017 *M*_*L*_5.4 earthquake and its aftershocks. (**a**) Map and cross sections of the mainshock and aftershocks. The earthquakes are divided into three groups. Vertical distribution of events for (**b**) group I along cross section AA’, (**c**) group I along cross section BB’, (**d**) group III along cross section BB’, and (**e**) group II along cross section CC’. The vertical distribution of aftershocks agrees with the focal mechanism solutions of major earthquakes. (**f**) Fault model and interaction between fault segments. The fault is divided into three fault segments at different depths. The figure was created using GMT 4.5.14 (https://www.soest.hawaii.edu/gmt/) and Adobe Illustrator CS6 (https://www.adobe.com/kr/products/illustrator.html).

The faults responsible for the three *M*_*L*_5-level earthquakes in 2016 and 2017 were not recognized before their occurrence. The 2016 *M*_*L*_5.1 and 5.8 earthquakes occurred in a strike-slip fault at depths between 11 and 16 km^22^. The dip of the fault was ~65° to the east. Additionally, the 2017 *M*_*L*_5.4 earthquake occurred in a blind crustal fault (Fig. 3). The aftershocks of the 2017 *M*_*L*_5.4 earthquake were distributed in a volume of 8 × 3 × 6 km^3^. The aftershock distribution and fault-plane solutions illuminate the geometry of the fault, which may be divided by three segments (Fig. 3).

Each fault segment developed at a different depth (Fig. 3). The focal depths of the earthquakes in the northeastern segment were shallow (4.5–7.2 km). On the other hand, most earthquakes in the southwestern segment were distributed at greater depths (≥6 km). The focal mechanism solutions of the earthquakes in the southwestern and northeastern segments indicate right-lateral strike-slip faults. By contrast, the focal mechanism solution of the *M*_*L*_4.3 earthquake in the central segment suggests a reverse faulting with a strike of N18°E and a dip of 62°. The seismic moment was 3.66 × 10^22^ Nm, and the corresponding moment magnitude was *M*_*w*_4.3.

The mainshock occurred at the boundary between the southwestern and central segments. The early sequence of aftershocks was located mainly in the southwestern segment, accompanying some aftershocks in the northeastern segment. Most of the aftershocks in the northeastern segment started to occur 2 hours after the mainshock (see supplementary materials). The later sequence of aftershocks was clustered around the central segment, which is located less than 2 km to the north of the mainshock. The earthquake migration suggests time-dependent faulting. Furthermore, the compression-axis directions of the 2017 *M*_*L*_5.4 earthquake and its aftershocks with magnitudes of *M*_*L*_ ≥ 3.6 for three months were determined to be similar (N80°W to N88°W). This observation suggests that the mainshock and aftershocks occurred under a constant stress environment.

The lateral distributions of aftershocks in the southwestern and northeastern segments are consistent with the strike of the mainshock. The fault plane in the southwestern segment dips to the NW at an angle of 61° from the surface. The fault plane of the northeastern segment developed at depths between 5.0 and 7.2 km. The dipping angle was 84°. The southwestern segment is connected to the northeastern segment by the central segment. The central segment plays the role of conjunction between the southwestern and northeastern segments. The reverse-faulting in the central segment may have been caused by lateral-stepwise motions of the northeastern and southwestern segments at different depths, inducing a vertical dislocation in the central segment (Fig. 3).

### Induction mechanism of time-advanced earthquakes

The crust of the Korean Peninsula was stretched by the differential coseismic and postseismic displacements due to the 2011 Tohoku-Oki megathrust earthquake, reducing the yield strengths in the crust^12^.

**Figure 4:**
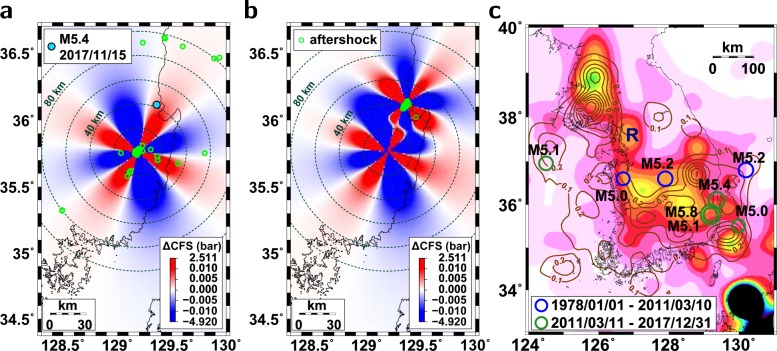
Coulomb stress changes and seismic hazard risks. (**a**) Coulomb stress changes at a depth of 10 km due to the 2016 *M*_*L*_5.8 earthquake, and (**b**) those additionally affected by the 2017 *M*_*L*_5.4 earthquake. The aftershocks of the 2017 *M*_*L*_5.4 earthquake are presented (circles). The 2017 *M*_*L*_5.4 earthquake occurred in a region of positive Coulomb stress change. The moderate-size earthquakes perturbed the local stress field. (**c**) Seismicity densities combined with instrumentally recorded earthquakes in 1978–2011 and historical earthquakes in 1392–1904^28^. The instrumental seismicity densities (contours) are presented for comparison^9^. The epicenters of major earthquakes since 1978 are marked. The earthquakes are not observed around the Seoul metropolitan area (region R). The figure was created using GMT 4.5.14 (https://www.soest.hawaii.edu/gmt/) and Adobe Illustrator CS6 (https://www.adobe.com/kr/products/illustrator.html).

The 2016 *M*_*L*_5.1 and *M*_*L*_5.8 earthquakes produced Coulomb stress changes of −4.9 to 2.5 bar for optimally oriented strike-slip faults at a depth of 10 km (Fig. 4). The induced Coulomb stress change for optimally oriented strike-slip faults at the location of the 2017 *M*_*L*_5.4 earthquake was 0.5 × 10^−2^ bar. The optimal orientation of right-lateral strike-slip faults was N41°E, which is close to the fault strike of the 2017 *M*_*L*_5.4 event (N45°E) (see supplementary materials). We found that the induced Coulomb stress change for the 2016 *M*_*L*_5.1 and *M*_*L*_5.8 earthquakes for the illuminated fault geometry of the 2017 *M*_*L*_5.4 earthquake was 0.2 × 10^−2^ bar at the hypocenter of the 2017 *M*_*L*_5.4 earthquake. The increased stress at the 2017 *M*_*L*_5.4 earthquake region was sufficiently larger than the critical stress change level (~1 × 10^−4^ bar) required to trigger earthquakes^30^.

No earthquakes with magnitudes greater than *M*_*L*_2.0 occurred in the region around the 2017 *M*_*L*_5.4 earthquake before the occurrence of the 2016 *M*_*L*_5.1 and *M*_*L*_5.8 earthquakes (see supplementary materials). The 2016 *M*_*L*_5.1 and *M*_*L*_5.8 earthquakes appeared to be the primary source of stress changes in the region of the 2017 *M*_*L*_5.4 earthquake. The 2017 *M*_*L*_5.4 earthquake occurred in a region of increased stress caused by the 2016 *M*_*L*_5.1 and *M*_*L*_5.8 earthquakes.

The 2017 *M*_*L*_5.4 earthquake caused additional stress changes, building a complex stress field around the southeastern Korean Peninsula (Fig. 4). The successive earthquakes in 2016–2017 perturbed the local stress field. The static stress in the interevent region between the 2016 *M*_*L*_5.8 earthquake and the 2017 *M*_*L*_5.4 earthquake was increased several fold by those earthquakes. Additionally, the 2016 and 2017 *M*_*L*_5-level earthquakes produced positive Coulomb stress changes at the offshore region in the direction NE of the 2017 *M*_*L*_5.4 earthquake.

## Discussion and Conclusions

Seismicity varies with stress^31,32^. Tectonic loading may be the primary source of the stress that has accumulated in the crust. The long-term tectonic-loading stress over the Korean Peninsula may be homogeneous. The 2011 *M*_*W*_9.0 Tohoku-Oki megathrust earthquake caused high perturbation in the medium and stress field. The induced stress change may play a crucial role in increasing the seismic hazard potential. A stress change may trigger earthquakes in regions of long-lived stress concentration^33,34^. The recent successive *M*_*L*_5-level earthquakes may be time-advanced events that are a consequence of medium weakening and stress perturbation due to the 2011 *M*_*W*_9.0 Tohoku-Oki megathrust earthquake and precedent adjacent events.

The recent inland *M*_*L*_5-level earthquakes occurred in crustal blind faults that were not identified before the events. Historically, large earthquakes have been recorded on the Korean Peninsula^8,9,35^. The recent increased seismicity with moderate-size earthquakes suggests high probability of hazardous-earthquake occurrence on the Korean Peninsula.

The spatial distribution of previous events may allow us to constrain the potential locations of future earthquakes. The seismicity density functions of instrumentally recorded and historical earthquakes may provide information on the stress released by precedent earthquakes (Figs 1 and 4). It is intriguing to note that the *M*_*L*_5-level earthquakes occurred in low seismicity regions (Fig. 1). The recent moderate-size earthquakes might occur around high prestressed regions with long-term cumulation of tectonic loading^36^. Faults in near-critical conditions might respond preferentially to additional stress changes induced by the 2011 Tohoku-Oki megathrust earthquake^32^. A major event releases the cumulated stress of the fault, triggering another event in adjacent regions.

The medium properties were recovered gradually with time, restoring the stress field. Several regions of high seismicity densities with low recent seismic activities exist (Fig. 4). The increased seismicity may continue until the medium properties and stress field are recovered. A combined interpretation of the instrumentally recorded and historical seismicity may suggest potential locations of devastating events that occurred historically (Fig. 4).

## Methods

The observed seismicity can be expressed as a sum of background seismicity and triggered seismicity^37^:

1 $$\lambda (x,t)={\lambda }_{0}(x)+\sum _{{t}_{i} < t}\,{\lambda }_{i}(x,t),$$

where *λ*(*x*, *t*) is the observed seismicity rate density at time *t* at location *x*, *λ*_0_(*x*) is the background intensity function, and *λ*_*i*_(*x*, *t*) is the contribution of earthquake *i* that occurred in time *t*_*i*_ at location *x*_*i*_. We decluster the earthquake catalog based on a nonparametric method that is useful for regions with low seismicity rates. The functions *λ*_*i*_(*x*, *t*) and *λ*_0_(*x*) are determined by an iterative process that assesses the numbers of event pairs in discrete bins of magnitudes, interevent times and distances^37–39^ (see supplementary materials).

We perform a long-period waveform inversion of earthquakes to determine the focal mechanism solutions^11,40^ based on a global-averaged one-dimensional (1-D) velocity model^41^. We determine a set of hypocentral parameters that generate the best-fit synthetic waveforms^42^. The focal mechanism solutions of the earthquakes are determined based on 5 seismic records of 0.05–0.1 Hz in regional distance (Fig. 2).

The hypocentral parameters of the earthquakes are refined using VELHYPO based on the *P* and *S* arrival times (see supplementary materials)^43,44^. This method is effective for the determination of hypocentral parameters of earthquakes in media with poorly known velocity structures (see supplementary materials). We implement a 1-D velocity model as the initial model^45^. We analyze 14 to 34 three-component waveforms of each earthquake for the hypocentral-parameter inversion. The average *P* and S travel time residuals are 6.1 × 10^−6^ s and 4.9 × 10^−3^ s, and their standard deviations are 0.0489 s and 0.1948 s, respectively (see supplementary materials). The horizontal and vertical location errors at the 95% confidence level are 9.7 m and 24.3 m. The origin time and hypocenter errors are sufficiently small.

The induced Coulomb stress change, ΔCFS can be represented by^46,47^

2 $${\rm{\Delta }}\mathrm{CFS}={\rm{\Delta }}\tau -\mu ^{\prime} \,{\rm{\Delta }}{\sigma }_{n},$$

where Δ*τ* is the shear stress change, *μ*′ is the effective frictional coefficient, and Δ*σ*_*n*_ is the normal stress change. We set the effective frictional coefficient *μ*′ to be 0.4^11,22,32,47,48^. The fault dimension is assumed to follow an empirical relationship with the seismic moment^49^. The ambient compressional stress field is oriented in the N77°E direction, with a strength of 65 bars^6,7,11,22^ (Fig. 1). In addition, we set the lithospheric Young’s modulus to be 80 GPa and the Poisson’s ratio to be 0.25^11,22,46,47^. The Coulomb stress changes induced by the earthquakes are calculated for media with strike-slip faults in the optimal orientation or given orientation^50^.

## Electronic supplementary material

Supplementary materials
