# Supplementary material for: Time-advanced occurrence of moderate-size earthquakes in a stable intraplate region after a megathrust earthquake and their seismic properties
Source: Sci Rep. 2018 Sep 6;8:13331. doi: 10.1038/s41598-018-31600-5 (PMC6127306; doi:10.1038/s41598-018-31600-5)
Supplement: Supplementary file 1 — Supplementary materials [file 41598_2018_31600_MOESM1_ESM.pdf]

Supplementary Materials for

Time-advanced occurrence of moderate-size earthquakes in a stable intraplate region after a megathrust earthquake and their seismic properties

Tae-Kyung Hong[a,*], Junhyung Lee[a], Seongjun Park[a], and Woohan Kim[b]

Affiliation and address:

[a]Yonsei University, Department of Earth System Sciences, 50 Yonsei-ro, Seodaemun-gu Seoul 120-749, South Korea.

[b]Gyeongsang National University, Department of Earth and Environmental Sciences and RINS, Jinju, Gyeongsangnam-do 660-701, South Korea.

Correspondence to:

tkhong@yonsei.ac.kr (Tae-Kyung Hong)

Seismicity declustering and seismicity change

The observed seismicity can be expressed as a sum of background and triggered seismicity (Zhuang et al., 2004; Marsan and Lengliné, 2008):

$$\lambda(x,t) = \lambda_0(x) + \sum_{t_i<t} \lambda_i(x,t), \tag{1}$$

where $\lambda(x,t)$ is the observed seismicity rate density in time t at location x, $\lambda_0(x)$ is the background seismicity intensity at location x, and $\lambda_i(x,t)$ is the contribution of earthquake i on location x in time t. The earthquake i occurred in time t_i at location x_i. We use a nonparametric method to determine the functions $\lambda_0(x)$ and $\lambda_i(x,t)$ that agree with the observed seismicity (Marsan and Lengliné, 2008). The nonparametric method is suitable for analysis of earthquake catalogs for regions with low seismicity rates.

The triggering effect of an earthquake may be dependent on the event magnitude, elapsed time, and distance. Thus, function $\lambda_i(x,t)$ can be expressed by (Marsan and Lengliné, 2008)

$$\lambda_i(x,t) = \lambda_t(t-t_i, m_i) \times \lambda_s(|x-x_i|, m_i), \tag{2}$$

where $\lambda_t(t-t_i, m_i)$ and $\lambda_s(|x-x_i|, m_i)$ are the temporal rates and normalized spatial densities of seismicity in time t at location x, which is triggered by event i with magnitude of m_i. The nonparametric triggered seismicity functions λ_t and λ_s can be assessed by an iteration process which investigate the number of event pairs within discrete bins of magnitude, interevent time and distance (Marsan and Lengliné, 2008).

We consider the discrete intervals of magnitude, time and distance to be 0.5 in magnitude unit, 0.2 in logarithmic time (days), and 0.25 in logarithmic distance (km). The iteration continues until the changes in logarithmic values of λ_0 and $\lambda_t \cdot \lambda_s$ are less than 1 %.

We collected the source information of earthquakes in and around the Korean Peninsula in 1978-2017 from the Korea Meteorological Administration (http://necis.kma.go.kr). The earthquake catalog is complete for events with magnitudes greater than or equal to 2.5 (Houng et al., 2013; Hong et al., 2015). We examine the minimum magnitude ($M_{\min}$) ensuring the completeness of the Korean earthquake catalog with four methods (Mignan and Woessner,

2012; Zhou et al., 2018) (Fig. S1). The minimum magnitudes are determined consistently ($M_{\min}$ =2.4, 2.5) among the methods.

We decluster the seismicity in the region of latitudes between 33°N and 40°N and longitudes between 124°E and 131°E. The number of events in the complete catalog is 919. The background and triggered seismicity functions λ_0, λ_t, and λ_s were calculated for the earthquake catalog (Fig. S2). The triggered seismicity appears to be proportional to the magnitude of mainshock, which is consistent with the general feature of aftershocks (Zhuang et al., 2004; Marsan and Lengliné, 2008).

The catalog is stochastically declustered based on the background and triggered seismicity functions. We identified 782 background earthquakes and 137 aftershock events (Fig. S3). The aftershocks were clustered around the mainshocks. We observed a large numbers of aftershocks for the 2016 M_L5.1 and 5.8 earthquakes and the 2017 M_L5.4 earthquake.

We determined the background seismicity rates for 365-days-long time windows that are shifted by 100 days. We observed rapid increase in the background seismicity rate after the 2011 M_W9.0 Tohoku-Oki megathrust earthquake.

It was reported that the occurrence of independent seismic events may follow the Poisson distribution in time (e.g., Gardner and Knopoff, 1974; Jafari, 2010; Wang, 2011). The Poisson probability is given by

$$P(n) = \frac{\mu^n}{n!} \exp[-\mu], \tag{3}$$

where $P(n)$ is the probability to have n-times event occurrence, and μ is the event-occurrence frequency.

Ten earthquakes with magnitudes greater than or equal to M_L5.0 occurred for 40 years in 1978-2018. The average occurrence rate of M_L5-level earthquakes for 40 years in 1978-2018 was 0.25 yr^{-1}, and that for 33 years in 1978-2010 (prior to the 2011 M_W9.0 Tohoku-Oki megathrust earthquake) was 0.15 yr^{-1}. The occurrence rate changed to 0.71 yr^{-1} since the megathrust earthquake.

The probabilities to have five M_L5-level earthquakes for 7 years in 2011-2018 (since the 2011 Tohoku-Oki megathrust earthquake) are estimated to be less than 3 % and 1 % in cases for average occurrence rates of 0.25 yr^{-1} and 0.15 yr^{-1}, respectively (Fig. S4). The observation

suggests that the recent M_L5-level earthquakes since the 2011 megathrust earthquake are statistically unusual.

Hypocentral-parameter inversion method: VELHYPO

We implement VELHYPO for hypocentral-parameter inversions of earthquakes (Kim et al., 2014; Kim et al., 2016). The method is effective for a hypocentral-parameter inversion of earthquake that occur in a region with poorly-known velocity structures.

VELHYPO combines a conventional hypocentral-parameter inversion method with a velocity-model refinement scheme. VELHYPO searches for an optimum 1-D velocity model yielding minimum misfit errors in hypocentral-parameter inversion. The number, thicknesses, and velocities of layers can be considered to be unknown.

A set of seismic velocity models are prepared by shifting the initial seismic velocity model by constant velocities (Kim et al., 2014; Kim et al., 2016):

$$\alpha_i^n = \alpha_i^0 + n\Delta\alpha, \quad (n = 0, 1, \cdots, N), \tag{4}$$

where $\Delta\alpha$ is a constant velocity interval, n is an integer varying from 0 to N, α_i^0 is the seismic velocity in the ith layer of the initial velocity model, and α_i^n is the seismic velocity in the ith layer of the nth velocity model.

Synthetic traveltimes of seismic phases are calculated for the velocity models. The misfit errors between the synthetic and observed traveltimes are calculated. We determine a semi-optimum velocity model that yields the minimum misfit error.

The semi-optimum velocity model is further refined by determining the optimum vertical velocity gradient. A set of velocity models modified from the semi-optimum velocity model is prepared. The seismic velocities in the layers are calculated by (Kim et al., 2014; Kim et al., 2016)

$$\alpha_i^{mod} = \alpha_i^{so} + (\alpha_1^{mod} - \alpha_1^{so}) \times \frac{\overline{\alpha} - \alpha_i^{so}}{\overline{\alpha} - \alpha_1^{so}}, \tag{5}$$

where α_i^{mod} is the modified seismic velocity for the ith layer, α_i^{so} is the seismic velocity for the ith layer in the semi-optimum model, and $\overline{\alpha}$ is the weighted average velocity of the velocity

model. The misfit errors for the set of velocity models are estimated. A velocity model with the minimum misfit error is selected as an optimum velocity model.

The 15 November 2017 M_L5.4 earthquake and aftershocks

We analyzed the 15 November 2017 M_L5.4 earthquake and its aftershocks until 31 December 2017. The number of events was 71. Seismic waveforms were collected from the seismic stations deployed in the Korean Peninsula (Fig. S5). The number of collected seismic waveforms was 37077. We analyzed 42-102 seismic waveforms for hypocentral-parameter inversions. The total number of seismic waveforms used for the hypocentral-parameter inversions was 5418. The hypocentral parameters were inverted using VELHYPO. We obtained seismic velocity models from the hypocentral-parameter inversions (Fig. S6). The inverted seismic velocity models are comparable among the events. The average velocity model generally agrees with a velocity model of an adjacent region (Kim, 1999).

The displacement spectra are calculated to examine the properties of radiated energy from earthquake. A theoretical displacement spectrum model, Ω, is given by (Brune, 1970)

$$\Omega(\omega) = \frac{M_0}{1 + (\omega/\omega_0)^2}, \tag{6}$$

where M_0 is the seismic moment, ω is the frequency, and ω_0 is the corner frequency. The displacement spectra at a common station (YOCB) in comparable distances are compared between the 12 September 2016 M_L5.8 earthquake and the 15 November 2017 M_L5.4 earthquake (Fig. S2). The hypocentral distances are 35.05 and 40.32 km. It is intriguing to note that the high frequency energy contents in horizontal components are larger than those in vertical component. The 12 September 2016 M_L5.8 earthquake displays high frequency contents compared to the 15 November 2017 M_L5.4 earthquake (Fig. S7).

We additionally perform long-period waveform inversions for the 15 November 2017 M_L5.4 earthquake and three aftershocks (the 15 November 2017 M_L4.3 earthquake, the 19 November 2017 M_L3.5 earthquake, and the 19 November 2017 M_L3.6 earthquake) (Dreger and Helmberger, 1990; Hong et al., 2015) (Figs. S8, S9). The earthquakes are observed to

be strike-slip events that are typical in the Korean Peninsula. The inverted focal mechanism solutions agree with the illuminated fault structure.

We refine the hypocentral parameters of earthquakes using VELHYPO (Kim et al., 2014; Kim et al., 2016). The epicenters of aftershocks for the 15 November 2017 M_L5.4 earthquake are relocated to be clustered around the illuminated fault plane (Fig. S10). The focal depths are refined to be shallower than the reported values of Korea Meteorological Administration (KMA) (Fig. S10). The misfit errors in the hypocentral-parameter inversion are small enough to certify the accuracy (Fig. S11).

The temporal evolution of aftershocks for the 15 November 2017 M_L5.4 earthquake suggests that the mainshock occurred in the southwestern segment (Fig. S12). The northeastern segment appears to be ruptured in 2 hours after the mainshock.

The strong motions produced by the 12 September 2016 M_L5.8 earthquake are comparable to those of the 15 November 2017 M_L5.4 earthquake (Figs. S13, S14). The spatial distribution of peak ground motions (PGAs) is similar between the events. The horizontal PGAs are stronger than vertical PGAs in both events.

The 2017 M_L5.4 earthquake occurred on a region of positive Coulomb stress changes that were induced by the 2016 M_L5-level earthquakes (Fig. S15). The optimal orientation of right-lateral strike-slip fault for the calculation of Coulomb stress changes is N41°E at the location of the 2017 M_L5.4 earthquake. The fault strike of The 2017 M_L5.4 earthquake is N45°E, which is close to the optimal orientation for peak Coulomb stress changes.

References

Brune, J. (1970). Tectonic stress and the spectra of seismic shear waves from earthquakes, Journal of Geophysical Research, 75, 4997-5009.

Dreger, D. S., and D. V. Helmberger (1990). Broadband modeling of local earthquakes, Bulletin of the Seismological Society of America, 80, 1162-1179.

Gardner, J.K., and Knopoff, L. (1974). Is the sequence of earthquakes in Southern California, with aftershocks removed, Poissonian?, Bulletin of the Seismological Society of America, 64 (5), 1363-1367.

Hong, T.-K., J. Lee, and S. E. Houng (2015). Long-term evolution of intraplate seismicity in stress shadows after a megathrust, Physics of the Earth and Planetary Interiors, 245, 59-70.

Houng, S.E., and Hong, T.-K. (2013). Probabilistic analysis of the Korean historical earthquake records, Bulletin of the Seismological Society of America, 103 (5), 2782-2796.

Ibáñez, J.M., De Angelis, S., Díaz-Moreno, A., Hernández, P., Alguacil, G., Posadas, A., and Pérez, N. (2012). Insights into the 2011-2012 submarine eruption off the coast of El Hierro (Canary Islands, Spain) from statistical analyses of earthquake activity, Geophysical Journal International, 191 (2), 659-670.

Jafari, M.A. (2010). Statistical prediction of the next great earthquake around Tehran, Iran, Journal of Geodynamics, 49, 14-18.

Kim, W. (1999). P-wave velocity structure of upper crust in the vicinity of the Yangsan Fault region, Geosciences Journal, 3 (1), 17-22.

Kim, W., T.-K. Hong, J. Lee, and T. A. Taira (2016). Seismicity and fault geometry of the San Andreas fault around Parkfield, California and their implications, Tectonophysics, 677, 34-44.

Kim, W., T.-K. Hong, and T.-S. Kang (2014). Hypocentral parameter inversion for regions with poorly known velocity structures, Tectonophysics, 627, 182-192.

Marsan, D., and O. Lengliné (2008). Extending earthquakes' reach through cascading. Science, 319, 1076-1079.

Mignan, A., and Woessner, J. (2012). Estimating the magnitude of completeness for earthquake catalogs, Community Online Resource for Statistical Seismicity Analysis, doi:10.5078/corssa-00180805.

Wang, Z. (2011). Seismic hazard assessment: issues and alternatives, Pure and Applied Geophysics, 168, 11-25.

Wiemer, S., and Wyss, M. (2000). Minimum magnitude of compelte reporting in earthquake catalogs: examples from Alaska, the western United States, and Japan, Bulletin of the Seismological Society of America, 90, 859-869.

Zhou, Y., Zhou, S., and Zhuang, J. (2018). A test on methods for MC estimation based on earthquake catalog, Earth and Planetary Physics, 2, 150-162.

Zhuang, J., Y. Ogata, and D. Vere-Jones (2004). Analyzing earthquake clustering features by using stochastic reconstruction. Journal of Geophysical Research, 109, B05301, doi:10.1029/2003JB002879.

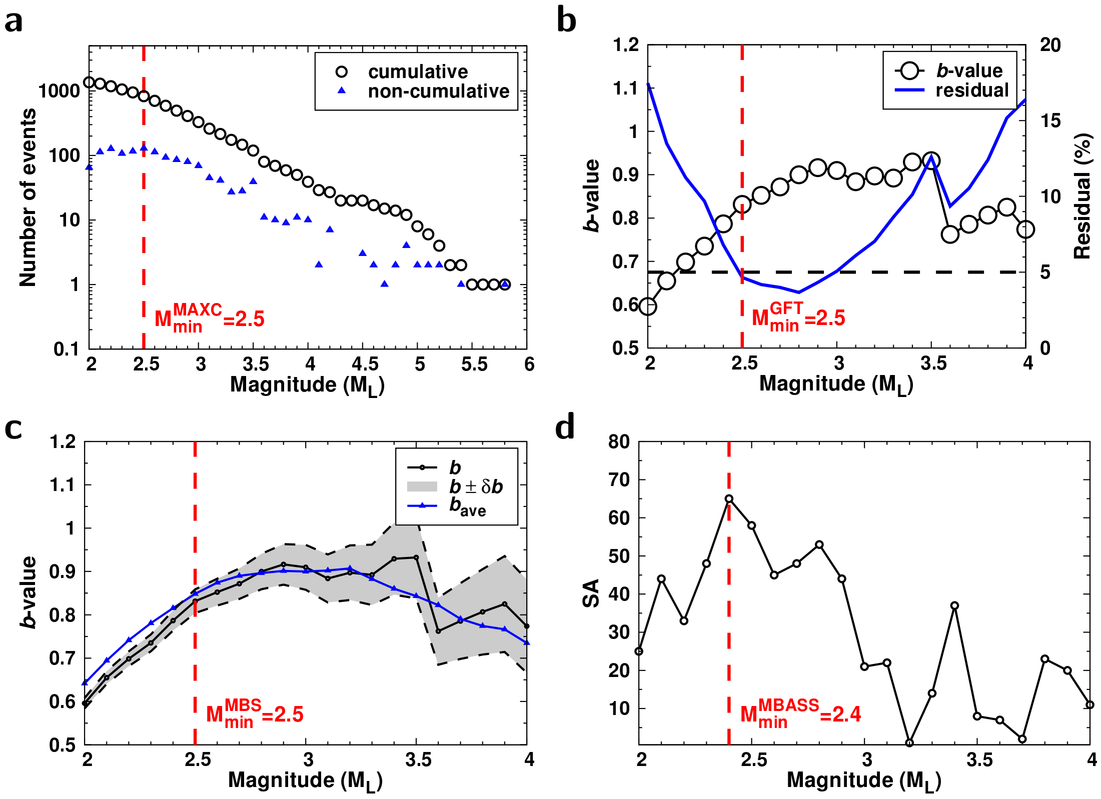

Figure S1. Estimation of the minimum magnitude ($M_{\min}$) for the Korean earthquake catalog with four methods including (a) the maximum curvature method (MAXC), (b) the goodness of fit method (GFT), (c) b-value stability method (MBS), and (d) the median-based analysis of the segment slope method (MBASS). The minimum magnitudes are estimated to be 2.4-2.5. The figure was created using Gnuplot 5.0 (https://www.gnuplot.info).

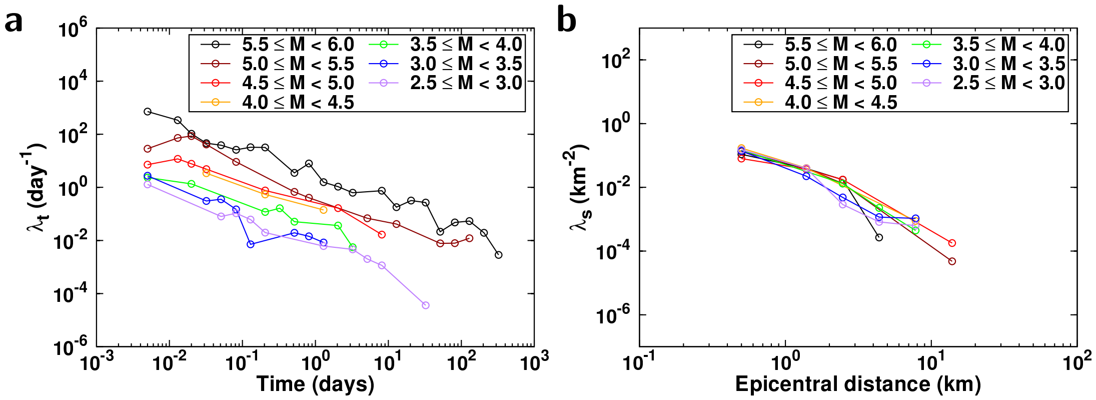

Figure S2. Triggered seismicity rates. (a) Temporal changes in triggered seismicity rates (λ_t) as a function of time in days and (b) normalized spatial densities of triggered seismicities (λ_s) as a function of distance in km. The triggered seismicity is observed to be proportional to mainshock magnitude, and decreases with time and distance. The figure was created using Gnuplot 5.0 (https://www.gnuplot.info).

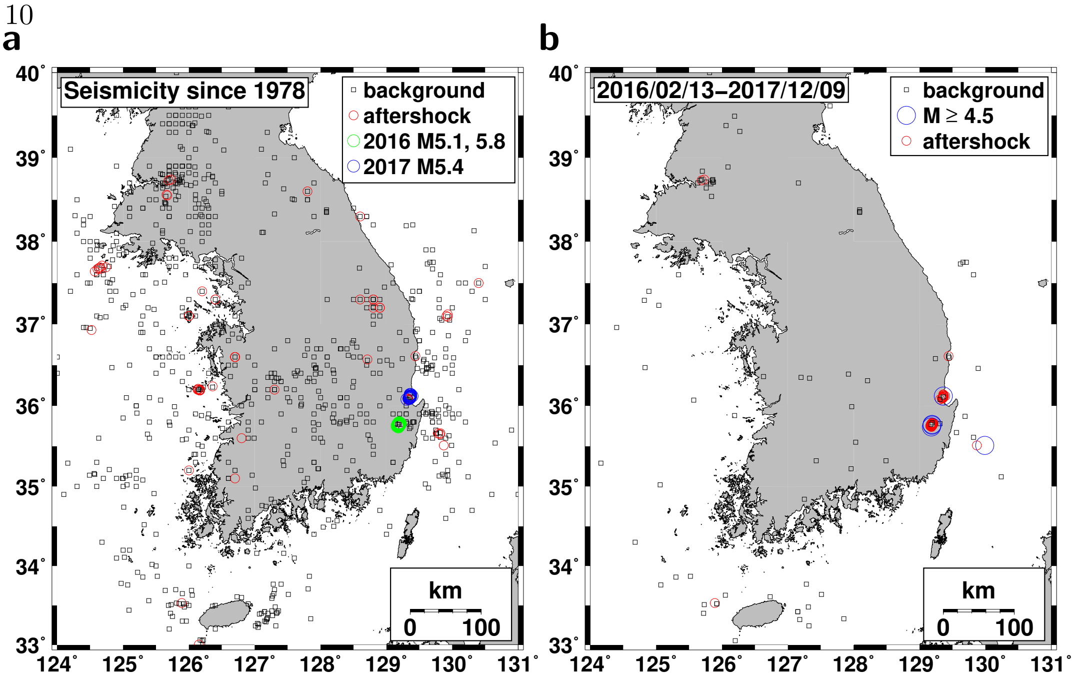

Figure S3. Background and triggered seismicity. (a) Declustered seismicity in 1978-2017. The background seismicity (squares) and aftershocks (circles) are marked. The aftershocks are observed to be clustered. The aftershocks of the 2016 M_L5.1 and 5.8 earthquakes and the 2017 M_L5.4 earthquake are well identified. (b) Declustered seismicity for a period of 13 February 2016 to 9 December 2017. The background seismicity appears to be independent from the moderate-size earthquakes in 2016 and 2017. The figure was created using GMT 4.5.14 (https://www.soest.hawaii.edu/gmt/).

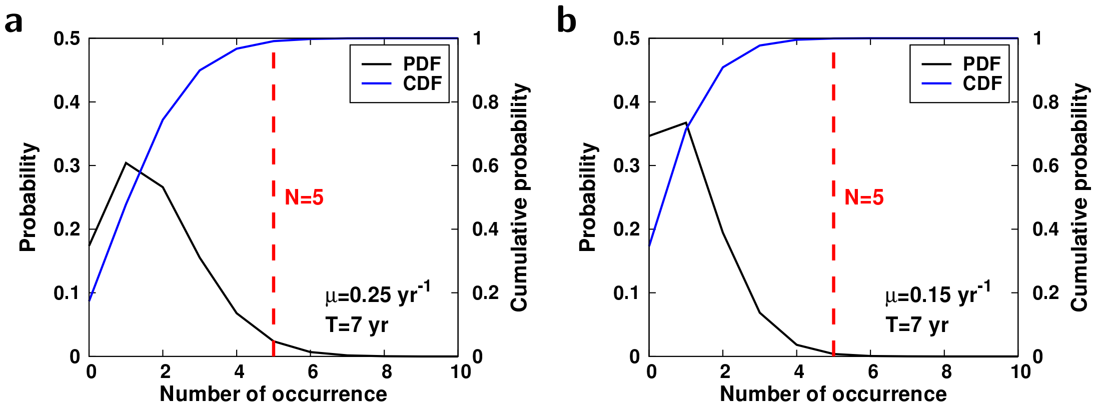

Figure S4. Probabilities to have 5 M_L5-level earthquakes for 7 years in 2011-2018 in Poissonian occurrence rates of (a) 0.25 yr^{-1} (10 M_L5-level earthquakes for 40 years in 1978-2018) and (b) 0.15 yr^{-1} (5 M_L5-level earthquakes for 33 years in 1978-2010). Probability density function (PDF) and cumulative density function (CDF) are presented. The probabilities to have 5 M_L5-level earthquakes for 7 years are marked with broken lines. The probabilities are less than 3 % and 1 %, respectively.

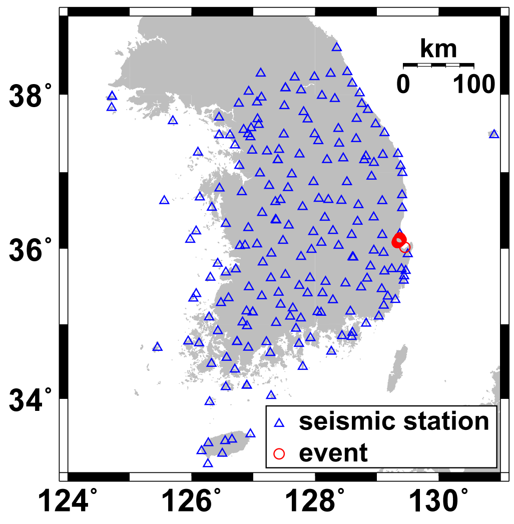

Figure S5. Spatial distribution of seismic stations (triangles) in the Korean Peninsula. The epicenters of 2017 M_L5.4 earthquake and aftershocks are marked with circles. The figure was created using GMT 4.5.14 (https://www.soest.hawaii.edu/gmt/).

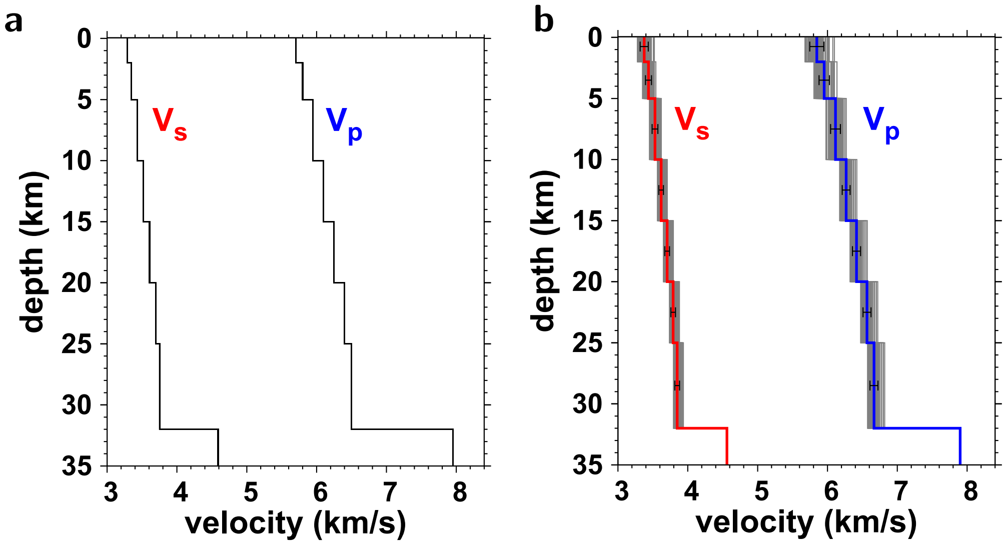

Figure S6. Seismic velocity models. (a) The initial model and (b) the inverted optimum velocity models for 71 events. The optimum velocity models were calculated from VELHYPO. The average velocity models and standard deviations are presented with thick solid lines and bars. The figure was created using GMT 4.5.14 (https://www.soest.hawaii.edu/gmt/) and Gnuplot 5.0 (https://www.gnuplot.info).

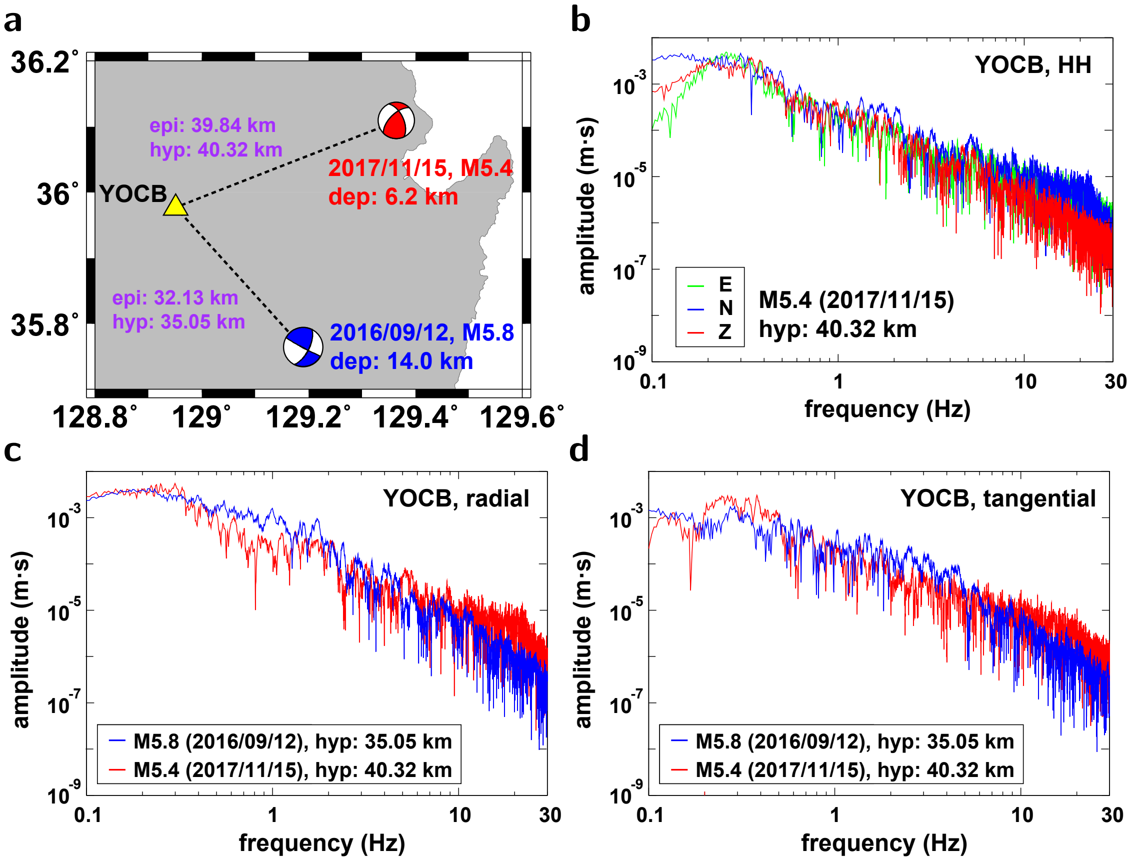

Figure S7. Comparison of displacement spectra at a station in comparable distances between the 12 September 2016 M_L5.8 earthquake and the 15 November 2017 M_L5.4 earthquake. (a) Map of earthquakes and station (YOCB). The focal depths and distances are denoted. (b) Displacement spectra of three components for the 15 November 2017 M_L5.4 earthquake. Comparison of (c) radial and (d) tangential displacement spectra between the earthquakes. The figure was created using GMT 4.5.14 (https://www.soest.hawaii.edu/gmt/) and Gnuplot 5.0 (https://www.gnuplot.info).

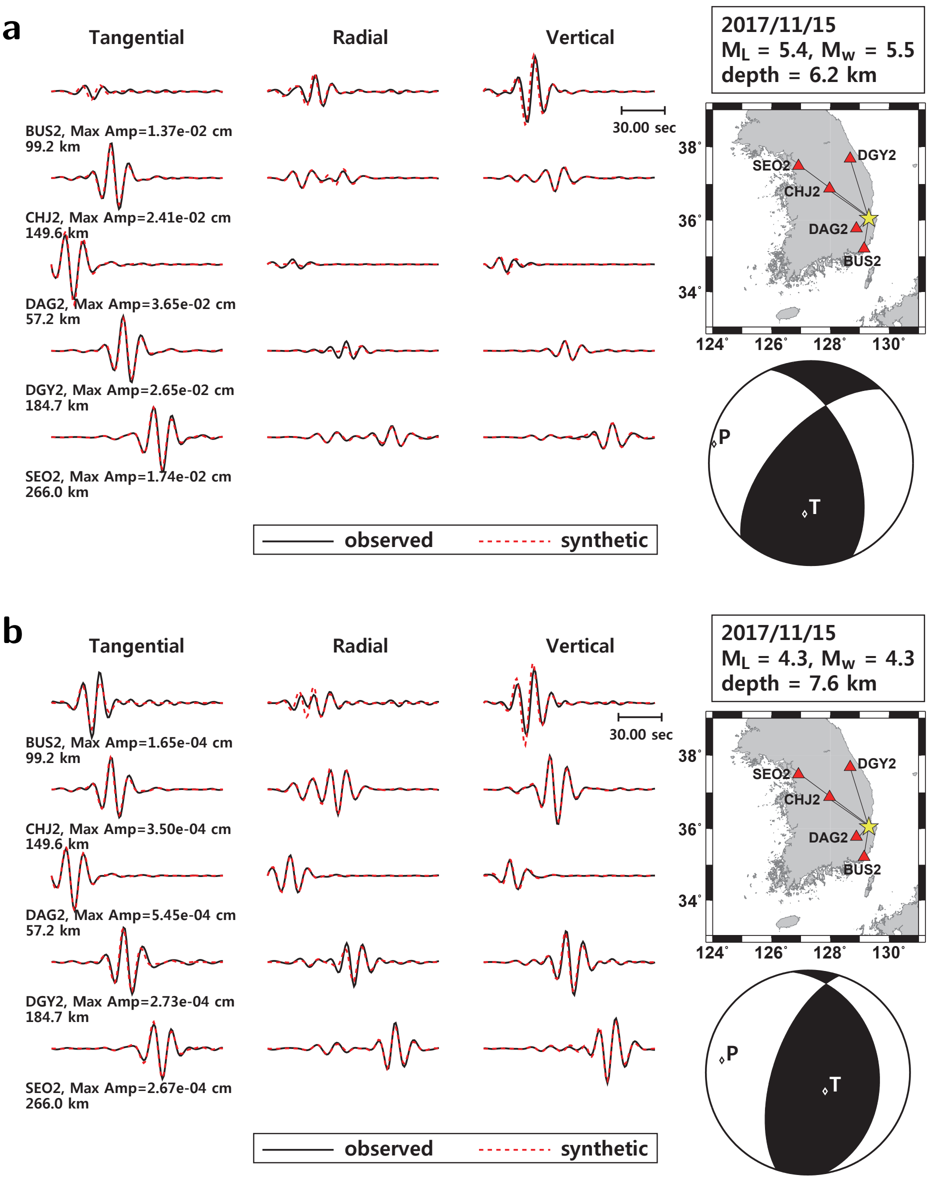

Figure S8. Long-period waveform inversion of (a) the 15 November 2017 M_L5.4 earthquake and (b) the 15 November 2017 M_L4.3 earthquake. The events display different types of faulting. The figure was created using GMT 4.5.14 (https://www.soest.hawaii.edu/gmt/) and Adobe Illustrator CS6 (http://www.adobe.com/kr/products/illustrator.html).

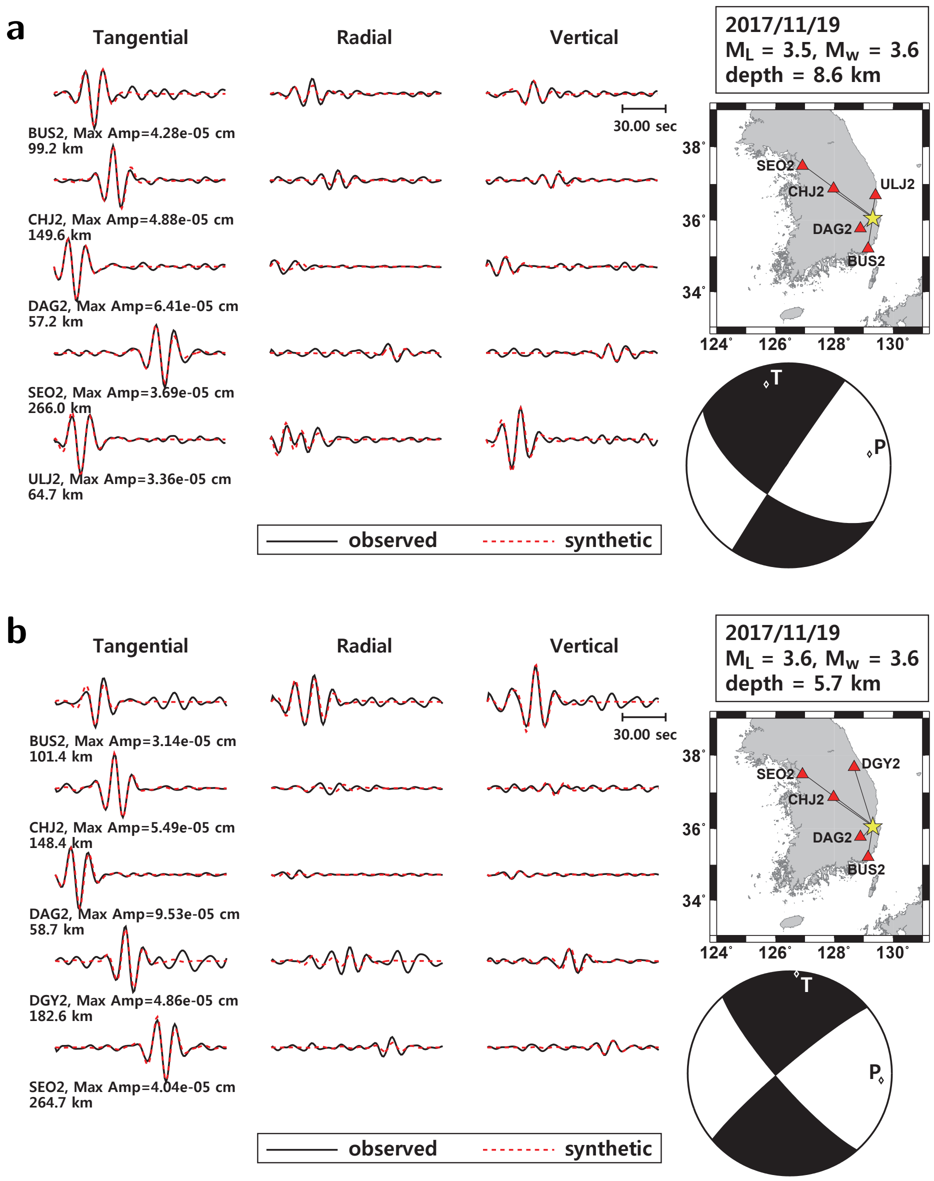

Figure S9. Long-period waveform inversion of two aftershock events of the 15 November 2017 M_L5.4 earthquake: (a) the 19 November 2017 M_L3.5 earthquake and (b) the 19 November 2017 M_L3.6 earthquake. The figure was created using GMT 4.5.14 (https://www.soest.hawaii.edu/gmt/) and Adobe Illustrator CS6 (http://www.adobe.com/kr/products/illustrator.html).

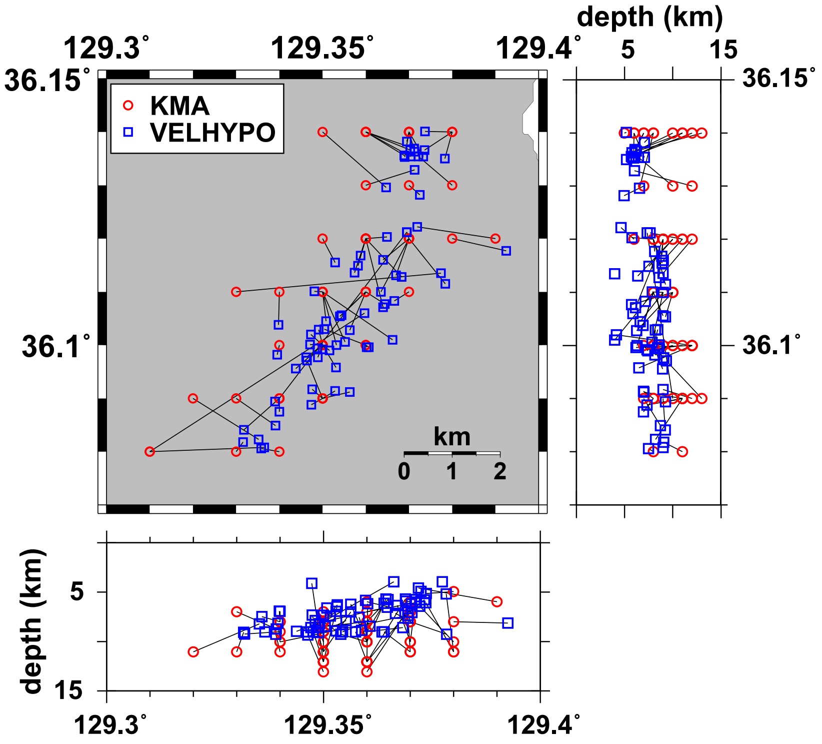

Figure S10. Relocation of aftershocks of the 15 November 2017 M_L5.4 earthquake. The event locations of Korea Meteorological Administration (KMA, circles) and the refined locations (squares) are interconnected. The refined focal depths are generally shallower than the reported focal depths. The figure was created using GMT 4.5.14 (https://www.soest.hawaii.edu/gmt/) and Adobe Illustrator CS6 (http://www.adobe.com/kr/products/illustrator.html).

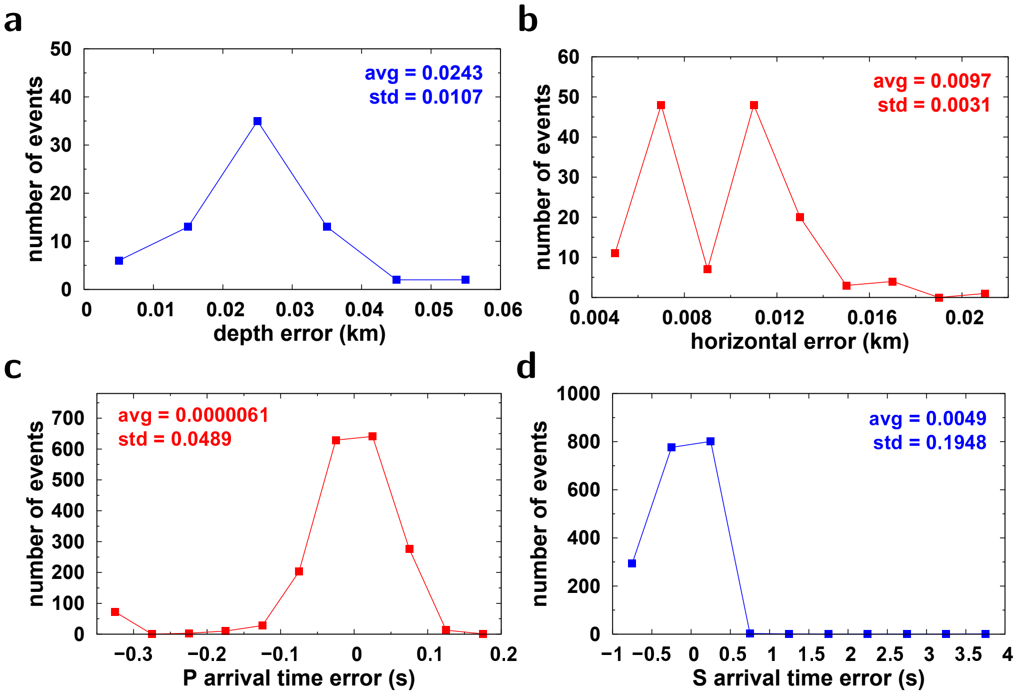

Figure S11. Misfit estimation in hypocentral-parameter inversion: (a) vertical location errors, (b) horizontal location errors, (c) P arrival time errors, and (d) S arrival time errors. The misfit amounts are generally small, supporting the accuracy. The figure was created using Gnuplot 5.0 (https://www.gnuplot.info) and Adobe Illustrator CS6 (http://www.adobe.com/kr/products/illustrator.html).

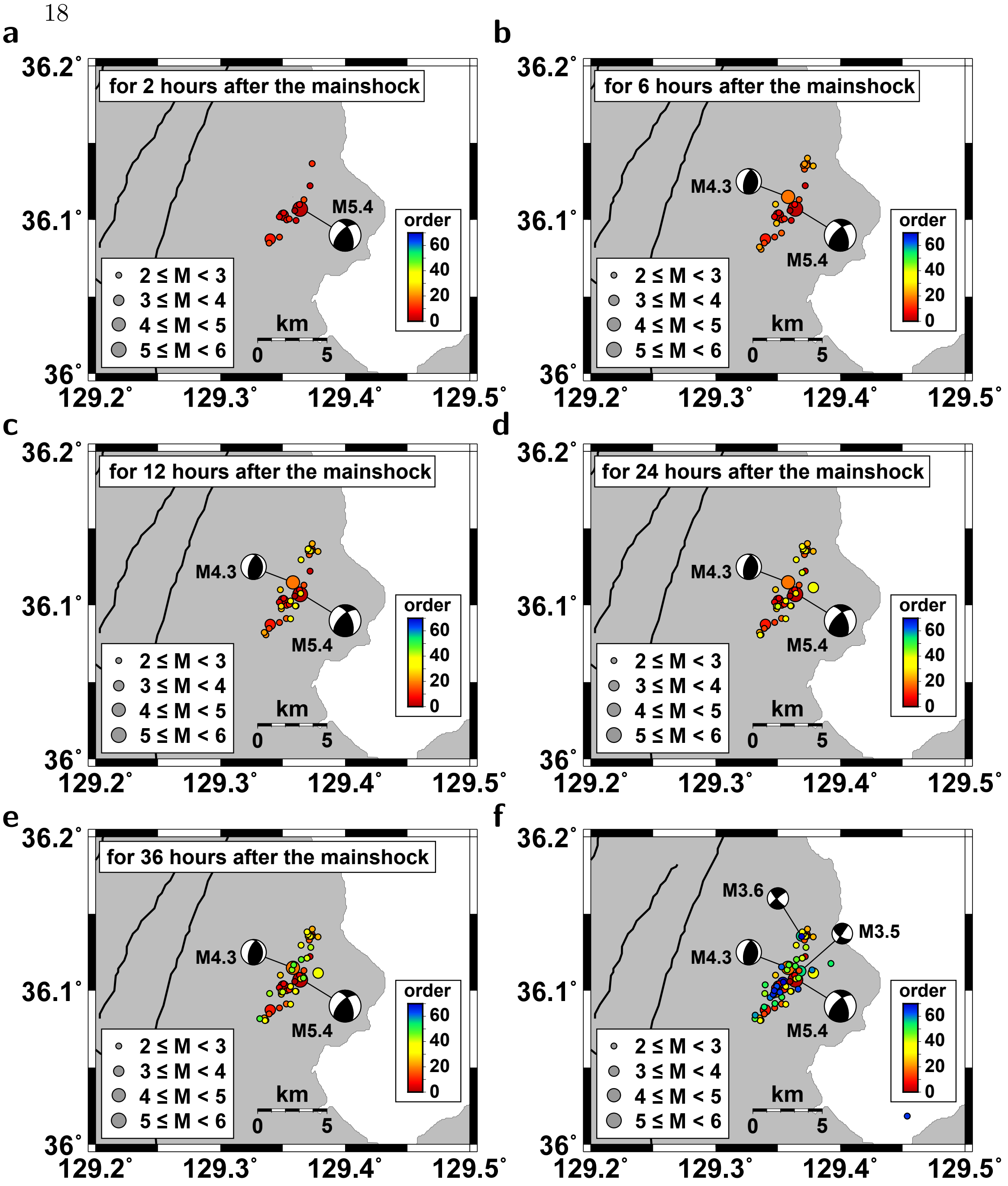

Figure S12. Aftershock sequence for (a) 2, (b) 6, (c) 12,, (d) 24, (e) 36 hours, and (f) 40 days after the 15 November 2017 M_L5.4 earthquake. The aftershocks were localized, and expanded with time. The figure was created using GMT 4.5.14 (https://www.soest.hawaii.edu/gmt/) and Adobe Illustrator CS6 (http://www.adobe.com/kr/products/illustrator.html).

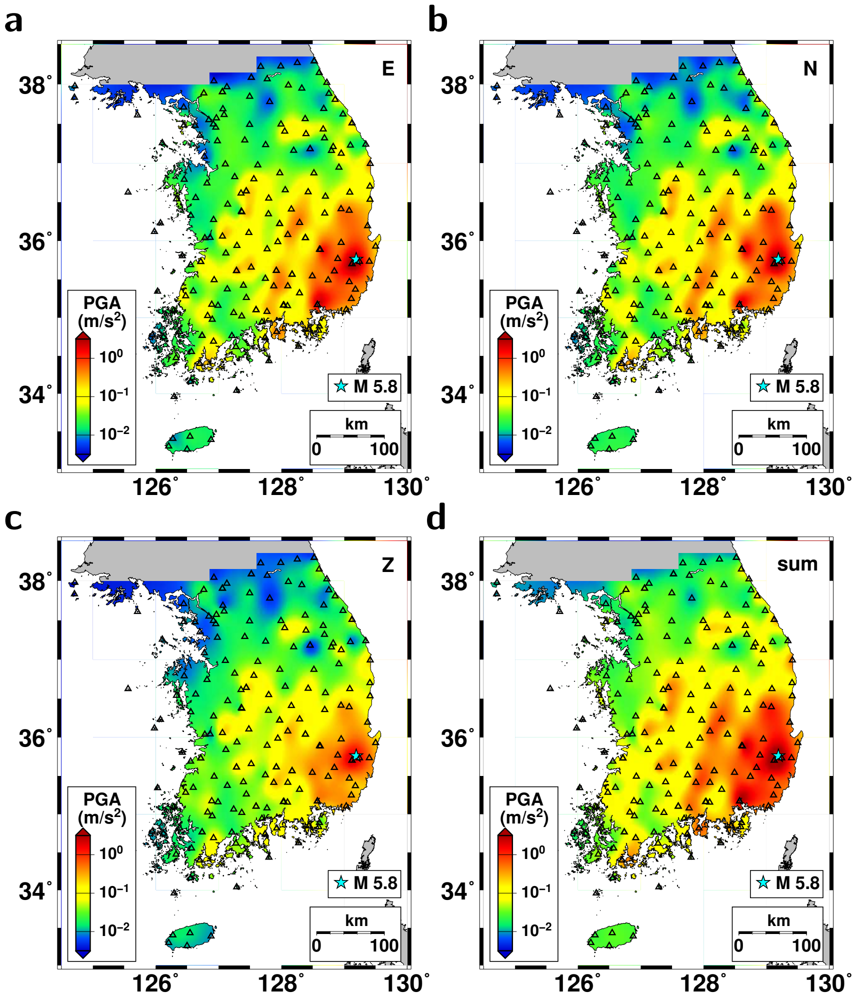

Figure S13. Peak ground accelerations (PGAs) of the 12 September 2016 M_L5.8 earthquake in (a) E-W, (b) N-S, (c) vertical components, and (d) vector sums. The horizontal PGAs are generally larger than the vertical PGAs. The figure was created using GMT 4.5.14 (https://www.soest.hawaii.edu/gmt/) and Adobe Illustrator CS6 (http://www.adobe.com/kr/products/illustrator.html).

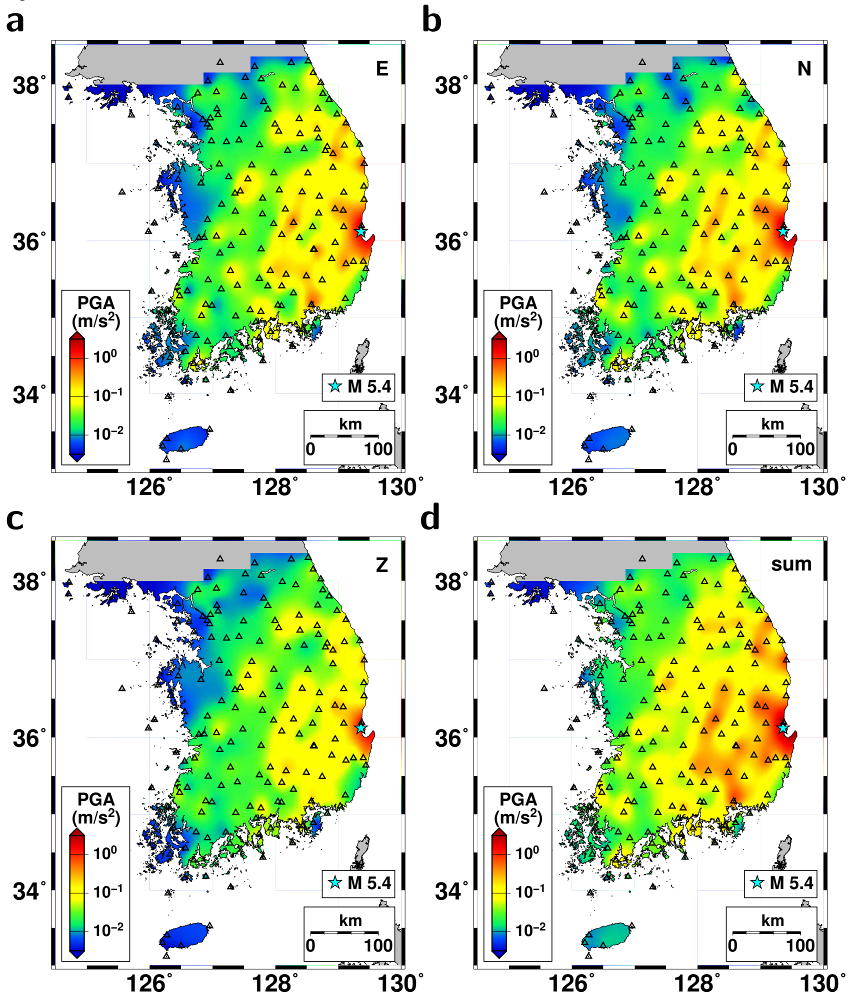

Figure S14. Peak ground accelerations (PGAs) of the 15 November 2017 M_L5.4 earthquake in (a) E-W, (b) N-S, (c) vertical components, and (d) vector sums. The horizontal PGAs are generally larger than the vertical PGAs. The figure was created using GMT 4.5.14 (https://www.soest.hawaii.edu/gmt/) and Adobe Illustrator CS6 (http://www.adobe.com/kr/products/illustrator.html).

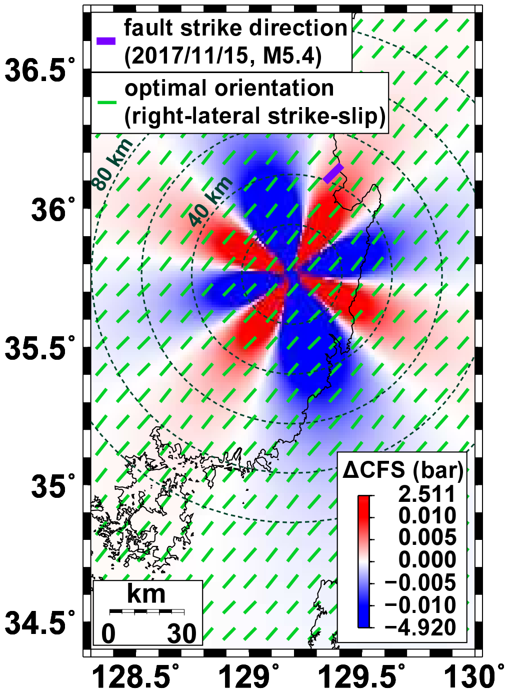

Figure S15. Coulomb stress changes induced by the 2016 M_L5.8 and 5.1 earthquakes. The optimal orientation of right-lateral strike-slip faults is indicated with thin bars. The fault strike of the 2017 M_L5.4 earthquake is presented with a thick bar. The figure was created using GMT 4.5.14 (https://www.soest.hawaii.edu/gmt/).
